# Ultrasound guided injection of botulinum toxin into the salivary glands of children with neurological disorders

**DOI:** 10.1590/0100-3984.2015.0056

**Published:** 2016

**Authors:** Marcia Wang Matsuoka, Sílvia Maria Sucena da Rocha, Lisa Suzuki, João Paulo Barnewitz, Rui Imamura, Luiz Antonio Nunes de Oliveira

**Affiliations:** 1 Hospital das Clínicas da Faculdade de Medicina da Universidade de São Paulo (HC-FMUSP), São Paulo, SP, Brazil.

*Dear Editor*,

Here, we report the case of a 2-year-old male patient with corpus callosum atrophy who was under investigation for genetic syndrome. The patient had a gastrostomy and a permanent tracheostomy. He had sialorrhea (drooling) that had not responded to clinical treatment with sublingual atropine and had been hospitalized for pneumonia on multiple occasions. He was referred for ultrasound-guided injection of botulinum toxin-recommended for therapeutic use since 1822^(1-7)^-into the parotid and submandibular glands.

**Figure 1:**
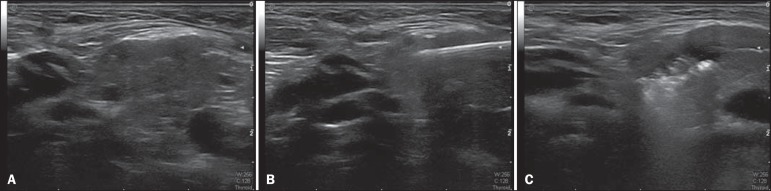
**A:** Normal right submandibular gland. **B:** Needle inserted into the gland. **C:** Botulinum toxin within the gland.

Ultrasound studies of the parotid and submandibular glands, all conducted by the same physician (with 15 years of experience in ultrasound), revealed that the glands were normal in appearance. Prior to, 30 days after, and 60 days after injection of the botulinum toxin, the glands were measured and their volumes were calculated. Ultrasound guidance allowed the best site for injection of the botulinum toxin to be identified, which prevented the toxin affecting structures adjacent to the salivary glands, such as the muscles involved in swallowing and vascular structures (Figure 1).

In follow-up visits, the mother reported that there was a significant decrease in the number of pads used for cleaning drool and a 50% reduction in the number of tracheal aspirations, without any complaints suggesting that the botulinum toxin had provoked an inflammatory process. The patient had no episodes of bronchopneumonia during the two-months observation period. The ultrasound studies of the parotid and submandibular glands showed no parenchymal changes subsequent to injection of the botulinum toxin.

The use of ultrasound to guide botulinum toxin injections is important in pediatric patients, especially because the small size of the salivary glands makes them difficult to palpate in such patients. In neurologically impaired children, the use of the ultrasound guidance is even more relevant, because they can present with increased muscle tone and often have a tracheostomy in an anatomically narrow location, as well as showing anatomical abnormalities^(1)^. In addition, the injection of botulinum toxin into adjacent structures could have undesirable effects, such as paralysis of the muscles involved in swallowing, which would worsen dysphagia^(1)^.

Previous studies have demonstrated that injection of botulinum toxin into the salivary glands does not cause any histological alterations-only lymphocyte infiltration, which results in homogeneous shrinkage of the gland without atrophy^(7)^. In addition, multiple injections of botulinum toxin over time can cause atrophy of the submandibular glands, thus promoting a permanent reduction in the severity of sialorrhea^(6)^. In the case presented here, we observed a reduction in the volume of all of the salivary glands injected, except the right parotid. We speculate that the injection was ineffective in that gland and that there was an increase in the volume of the gland through vicarious mechanisms. The study of glandular volume in such cases is groundbreaking, and our group is contemplating further studies in this line of reasearch. In the literature, we found no articles comparing glandular dimensions before and after botulinum toxin injection in neurologically impaired children. A study conducted by Cardona et al.^(8)^ showed no differences in glandular dimensions between children with and without sialorrhea. We seek to disseminate the knowledge that ultrasound guidance makes the injection of botulinum toxin into the salivary glands safer and more precise, especially in pediatric patients, as well as that ultrasound represents a noninvasive method of evaluating changes in the volume of those glands over time.
